# Resultados Clínicos e Hemodinâmicos de Longo Prazo após o Transplante de Coração em Pacientes Pré-Tratados com Sildenafil

**DOI:** 10.36660/abc.20190047

**Published:** 2021-02-19

**Authors:** Sofia Lázaro Mendes, Nadia Moreira, Manuel Batista, Ana Rita Ferreira, Ana Vera Marinho, David Prieto, Rui Baptista, Susana Costa, Fatima Franco, Mariano Pego, Manuel de Jesus Antunes

**Affiliations:** 1 Centro Hospitalar e Universitario de Coimbra EPECoimbraPortugalCentro Hospitalar e Universitario de Coimbra EPE, Coimbra - Portugal

**Keywords:** Resistência Vascular, Transplante de Coração, Hipertensão Pulmonar, Citrato de Sildenafila, Inibidores da Fosfodiesterase 5, Disfunção Ventricular Direita

## Abstract

**Fundamento:**

A resistência vascular pulmonar elevada ainda é um grande problema na seleção de candidatos ao transplante cardíaco.

**Objetivo:**

Nosso objetivo foi avaliar o efeito da administração de sildenafila pré-transplante cardíaco em pacientes com hipertensão pulmonar fixa.

**Métodos:**

O estudo retrospectivo, de centro único, incluiu 300 candidatos a transplante cardíaco consecutivos tratados entre 2003 e 2013. Destes, 95 pacientes tinham hipertensão pulmonar fixa e, dentre eles, 30 pacientes foram tratados com sildenafila e acabaram passando pelo transplante, formando o Grupo A. O Grupo B incluiu 205 pacientes sem hipertensão pulmonar que passaram pelo transplante cardíaco. A hemodinâmica pulmonar foi avaliada antes do transplante, 1 semana e 1 ano após o transplante. A taxa de sobrevivência foi comparada entre os grupos. Neste estudo, um P valor < 0,05 foi considerado estatisticamente significativo.

**Resultados:**

Após o tratamento com sildenafila, mas antes do TxC, a RVP (-39%) e a PAPs (-10%) diminuíram significativamente. A PAPs diminuiu após o TxC em ambos os grupos, mas permaneceu significativamente alta no grupo A em relação ao grupo B (40,3 ± 8,0 mmHg versus 36,5 ± 11,5 mmHg, P=0,022). Um ano após o TxC, a PAPs era 32,4 ± 6,3 mmHg no Grupo A versus 30,5 ± 8,2 mmHg no Grupo B (P=0,274). O índice de sobrevivência após o TxC 30 dias (97% no grupo A versus 96% no grupo B), 6 meses (87% versus 93%) e um ano (80% versus 91%) após o TxC não foi estatisticamente significativo (Log-rank P=0,063). Depois do primeiro ano, o índice de mortalidade era similar entre os dois grupos (sobrevivência condicional após 1 ano, Log-rank p=0,321).

**Conclusão:**

Nos pacientes com HP pré-tratados com sildenafila, a hemodinâmica pós-operatória inicial e o prognóstico são numericamente piores em pacientes sem HP, mas depois de 1 ano, a mortalidade em médio e longo prazo são semelhantes. (Arq Bras Cardiol. 2021; 116(2):219-226)

## Introdução

O transplante cardíaco (TxC) é o padrão ouro do cuidado da insuficiência cardíaca terminal.^1^ Estudos epidemiológicos mostraram que 60-70% dos pacientes com insuficiência cardíaca (IC) desenvolvem hipertensão pulmonar (HP).^2,3^ Em um estudo da Mayo Clinic,^4^ detectou-se uma forte associação graduada entre pressão arterial pulmonar sistólica (PAPs) e mortalidade e, por isso, a presença de HP grave é uma das maiores contraindicações ao TxC, devido à disfunção do coração direito pós-operatória.^5^

Pressões elevadas no lado direito em IC geralmente resultam de pressões de enchimento elevadas no ventrículo esquerdo (VE). Portanto, a pressão arterial pulmonar diastólica (PAPd) está firmemente correlacionada com a pressão capilar pulmonar (PCP).^6,7^ Por outro lado, o componente vasorreativo da HP se desenvolve com a HP duradoura. Ele é caracterizado por vasoespasmo, vasoconstrição e alterações morfológicas dos vasos.^8,9^Nesse caso, a HP persiste, independentemente da diminuição da PCP após o TxC. Refletindo os componentes “fixos” da HP, a resistência vascular pulmonar (RVP) e o gradiente transpulmonar (GTP) são elevados.^6^

A princípio, a HP é reversível por vasodilatadores sistêmicos, mas, posteriormente ela se torna relativamente estática ou “fixa”.^6,9,10^A RVP elevada aumenta a mortalidade no período inicial pós-TxC e continua sendo um grande problema para a seleção dos candidatos.^11,12^ A impossibilidade do coração transplantado de se adaptar a HP significativa pré-existente geralmente resulta em insuficiência do ventrículo direito (VD), que representa aproximadamente 50% de todas as complicações cardíacas e até 19% de todas as mortes precoces no pós-operatório.^12,13^Por isso, a avaliação correta que a reatividade do sistema vascular pulmonar exerce para a terapia vasodilatadora tem um papel crucial na seleção do candidato. As diretrizes da American Heart Association definem HP fixa como média da pressão arterial pulmonar (mPAP) ≥ 25 mmHg e RVP ≥ 2,5 unidades Wood (UW) e/ou GTP ≥ 12 mmHg, mesmo depois da testagem com vasodilatador farmacológico.^14^

A sildenafila é um inibidor de fosfodiestarase tipo 5 (PDE5) seletivo e potente que, especificamente, degrada a guanosina monofosfato cíclico, o segundo mensageiro do óxido nítrico nas células musculares lisas vasculares.^8,15^ A sildenafila tem um perfil favorável sem desnaturação do oxigênio ou alterações significativas da frequência cardíaca ou da pressão sanguínea.^16^ Vários estudos de centro único demonstraram efeito hemodinâmico favorável da administração da sildenafila pré-TxC em candidatos ao TxC com HP.^12,17^ Entretanto, há uma escassez de dados sobre os resultados iniciais e de longo prazo sobre esses pacientes de alto risco.

O objetivo deste estudo é comparar o efeito na hemodinâmica inicial do VD e mortalidade após o TxC da administração da sildenafila pré-TxC em pacientes com HP fixa que se qualificaram para o TxC e em pacientes sem HP. Nossa hipótese é de que os pacientes com HP que foram transplantados com o uso de sildenafila têm prognósticos comparáveis aos dos pacientes sem HP.

## Métodos

### População do Estudo

Este estudo de observação, de centro único e retrospectivo incluiu 300 pacientes consecutivos, candidatos a TxC, observados entre novembro de 2003 e dezembro de 2013. A população incluiu 95 pacientes com hipertensão pulmonar fixa. Dentre eles, 30 pacientes foram tratados com sildenafila e acabaram passando pelo transplante, formando o Grupo A. O Grupo B incluiu 205 pacientes sem HP fixa que passaram pelo TxC.

No grupo A, a sildenafila foi administrada via oral a 20 mg t.i.d., durante um período médio de 65 dias (faixa 4 - 81) antes do TxC. A sildenafila foi bem tolerada em todos os pacientes envolvidos, sem que eventos adversos sérios tivessem sido observados.

### Coleta de Dados

Dados clínicos, laboratoriais e hemodinâmicos foram extraídos usando um software dedicado. Todos os pacientes passaram por um cateterismo do coração direito (CCD) com um cateter Swan-Ganz, pela veia femoral, antes do começarem a usar a sildenafila. O grupo de pacientes que foram expostos à sildenafila passaram por um segundo CCD para avaliar o efeito hemodinâmico da droga. Depois do TxC, as pressões sistólica do ventrículo direito e diastólica final foram registradas durante a primeira biópsia endomiocárdica, que foi realizada 1 semana após o TxC. Um acompanhamento hemodinâmico tardio foi coletado durante o CCD pré-definido 1 ano após o TxC em ambos os grupos.

O débito cardíaco (DC) foi medido pelo método de Fick, e o índice cardíaco (IC) foi calculado dividindo-se o DC pela área da superfície do corpo. A PCP, a PAPs, a PAPd e a mPAP foram medidas automaticamente. RVP e GTP foram calculados utilizando-se as seguintes fórmulas: GTP (mmHg) = mPAP - PCP; RVP (UW) = GTP/DC.^18^ Um acompanhamento foi realizado por um período médio de 6,9 anos (faixa 4,2 - 6,9 anos) por entrevista pessoal na clínica, análise de registros hospitalares e contato telefônico, e foi realizado para todos os pacientes incluídos. A confidencialidade foi sempre respeitada.

### Endpoints

As medidas de resultados coprimários foram (1) Pressão sistólica de VD e pressão diastólica final (a última usada como substituta da função VD) 7 dias após o TxC e (2) a PAPs e RVP, 1 ano após o TxC. O resultado secundário foi a mortalidade global após o TxC. Os endpoints forma comparados entre grupos pré-definidos.

### Análise Estatística

Variáveis contínuas foram distribuídas normalmente e avaliado usando-se o teste de Shapiro-Wilk, e expressas como média ± desvio padrão, e as com distribuição não normal foram expressas como média (faixa interquartil). Variáveis dicotômicas foram expressas como frequências (porcentagens). Para comparar dados entre os grupos, utilizamos o teste T de Student (teste T não pareado) para variáveis contínuas, teste Mann-Whitney para dados não contínuos, e teste qui-quadrado (Fisher, conforme apropriado) para dados dicotômicos. O teste de McNemar foi usado para análise de dados categóricos pareados. As curvas de sobrevivência de Kaplan-Meyer foram construídas e comparadas usando o teste Log-rank. A sobrevivência condicional foi avaliada limitando-se o grupo de pacientes analisado aos que sobreviveram pelo menos 1 ano. A análise total foi realizada utilizando-se o software STATA 12.0 (College Station, Texas, EUA). Os gráficos foram construídos com o software GraphPad 5.0 (La Jolla, California, EUA). Neste estudo, um P valor < 0,05 foi considerado estatisticamente significativo.

## Resultados

Todos os 235 pacientes passaram por TxC com sucesso. As características da linha de base são apresentadas na Tabela 1. A maioria dos pacientes era do sexo masculino, e a média de idade do grupo A foi de 53,6 ± 10,9 anos, e do grupo B 52,9 ± 13,4 anos (p = 0,545). A hemodinâmica pré-TxC é apresentada na Tabela 2 e foi significativamente diferente entre os grupos. Os pacientes do grupo A apresentaram hemodinâmica pulmonar mais grave do que os pacientes do grupo B. Após o tratamento com sildenafila, mas antes do TxC, o RVP (-39%) e a PAPs (-10%) diminuíram significativamente (Tabela 3).

**Tabela 1:** – Características de pacientes com (Grupo A) e sem (Grupo B) pré-tratamento com sildenafila antes do transplante cardíaco

Característica ^**a**^	Grupo A (n=30)	Grupo B (n = 205)	Valor de p ^**b**^
Média de idade, anos	53,6 ± 10,9	52,9 ± 13,4	0,545
Sexo masculino, %	86,7	76,2	0,247
**Etiologia**			
Isquêmica, %	50,0	34,0	0,346
Idiopática, %	36,7	56,3	
Hipertrófica, %	3,3	4,4	
Restritiva, %	10,0	2,9	
Congênita, %	0,0	2,4	
**Classe NYHA**			
III, %	33,3	36,4	0,968
IV, %	66,7	63,6	
**Parâmetros laboratoriais**			
Hemoglobina, g/dl	12,3 ± 1,8	12,7 ± 1,7	0,815
Creatinina, mg/dl	1,4 ± 1,0	1,3 ± 0,5	0,060
BNP, pg/ml	524 [396 - 912]	625 [306 - 1039]	0,906
**Parâmetros cardíacos**			
FEVE, %	19,6 ± 4,5	21,2 ± 8,4	0,021
**Regurgitação mitral**			
Leve, %	16,0	12,5	0,703
Moderada, %	32,0	34,0	
Moderada a Grave, %	24,0	14,6	
Grave, %	24,0	31,2	
**Dispositivos cardíacos**			
CDI, %	40,0	21,8	0,128
CRT, %	10,0	22,4	
**Sildenafila, pré-TxC**			
Duração, dias	65 [4 – 181]		

BNP: peptídeo natriurético do tipo B; TRC: terapia de ressincronização cardíaca; TxC: transplante cardíaco; CDI: cardioversor desfibrilador implantável; FEVE: fração de ejeção do ventrículo esquerdo; NYHA: New York Heart Association. ^*a*^Os dados são expressos como porcentagens, média ± desvio padrão ou média (faixa interquartil).^* b*^Teste T de Student para variáveis contínuas com distribuição normal, teste de Mann-Whitney para variáveis contínuas sem distribuição normal, e Teste qui-quadrado para variáveis categóricas.

**Tabela 2:** – Variáveis hemodinâmicas antes do transplante cardíaco em pacientes com (Grupo A) e sem (Grupo B) hipertensão grave

Variável	Grupo A Média ± DP	Grupo B Média ± DP	Valor de p a
RVP, UW	5,4 ± 2,3	2,7 ± 1,8	<0,001
**PAPs (mmHg)**			
Sistólica	58,9 ± 16,4	44,5 ± 15,2	<0,001
Diastólica	23,1 ± 8,2	19,4 ± 8,0	0,025
Média	36,4 ± 10,7	29,0 ± 10,3	0,001
DC, litros/min	3,7 ± 1,2	3,6 ± 1,0	0,645
**PS, mmHg**			
Sistólica	75,0 ± 12,2	74,9 ± 10,8	0,980
FC, ppm	76 ± 18	76 ± 16	0,873

PS: pressão sanguínea; FC: frequência cardíaca; DC: débito cardíaco; PAP: pressão arterial pulmonar; RVP: resistência vascular pulmonar; DP: desvio padrão. aFoi usado o teste T de Student.

**Tabela 3:** – Variáveis hemodinâmicas antes e depois do Transplante cardíaco em pacientes com (Grupo A) e sem (Grupo B) pré-tratamento com sildenafila

	Qualificação para CCD		CCD 3-meses após sildenafila		BEM 7 dias após o TxC		CCD de 1 ano	
	PAPs (mmHg)	RVP (UW)	PAPs (mmHg)	RVP (UW)	VD sistólica pressão (mmHg)	VD diastólica final pressão (mmHg)	PAPs (mmHg)	RVP (UW)
**Sem sildenafila**	44,5 (15,2)	2,7 (1,8)	--	--	36,5 (11,5)	7,0 (7,1)	30,48 (8,23)	1,8 (1,0)
**Sildenafila**	58,9 (16,4)	5,4 (2,3)	52,8 (17,1)^c^	3,3 (2,3)^d^	40,3 (8,0)	7,9 (5,8)	32,43 (6,39)	1,8 (0,8)
**Valor de p**^**a**^	< 0,001	< 0,001	--	--	0,022^b^	0,374^b^	0,274	0,789

BEM: biópsia endomiocárdica; RVP: resistência vascular pulmonar; CCD: cateterismo do coração direito; UW: unidades Wood. ^*a Teste*^ t de Student comparando pacientes sem sildenafila pacientes a pacientes tratados com sildenafila. ^*b*^Teste de McNemmar. ^*c*^p = 0,845 versus pacientes sem sildenafila. ^*d*^p = 0,806 versus pacientes sem sildenafila.

### Dados Peri-TxC e Resultados Pós-TxC

**Figura 1:**
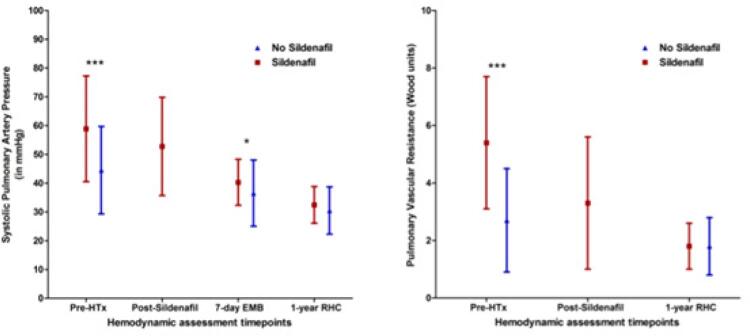
– (Painel esquerdo) Pressão arterial pulmonar sistólica (PAPs, em mmHg) em quatro momentos diferentes: linha de base antes do transplante cardíaco (TxC) sem tratamento com sildenafila, antes do TxC com tratamento com sildenafila, logo após o TxC (7 dias) e muito tempo após o TxC (um ano). ***p < 0,001, * p = 0,022. (Painel direito) Resistência vascular pulmonar (RVP, em unidades Wood) em três pontos no tempo diferentes: linha de base antes do (TxC) sem tratamento com sildenafila, antes do TxC com tratamento com sildenafila, e mais tarde após o TxC (um ano). *** p < 0,001, * p = 0,789. BEM: biópsia endomiocárdica; TxC: transplante cardíaco; CCD: cateterismo do coração direito.

As medidas de endpoints coprimários, avaliadas 1 semana após o TxC, são apresentadas na Tabela 3. A evolução da PAPs durante o período de acompanhamento em ambos os grupos é apresentada na Figura 1. A PAPs diminuiu após o TxC em ambos os grupos, mas permaneceu significativamente alta no grupo A em relação ao grupo B (40,3 ± 8,0 mmHg versus 36,5 ± 11,5 mmHg, p=0,022). Não foram encontradas diferenças em relação à pressão diastólica final do VD uma semana após o TxC, usado como substituo da disfunção de VD inicial (Tabela 3). Um ano após o TxC, a PAPs era 32,4 ± 6,3 mmHg no Grupo A versus 30,5 ± 8,2 mmHg no Grupo B (*P*=0,274) (Tabela 3). A RVP também foi semelhante entre os dois grupos (1,8 ± 0,8 mmHg versus 1,8 ± 1,0 UW, p = 0,789).

### Análise dos Índices de Sobrevivência

**Figura 2:**
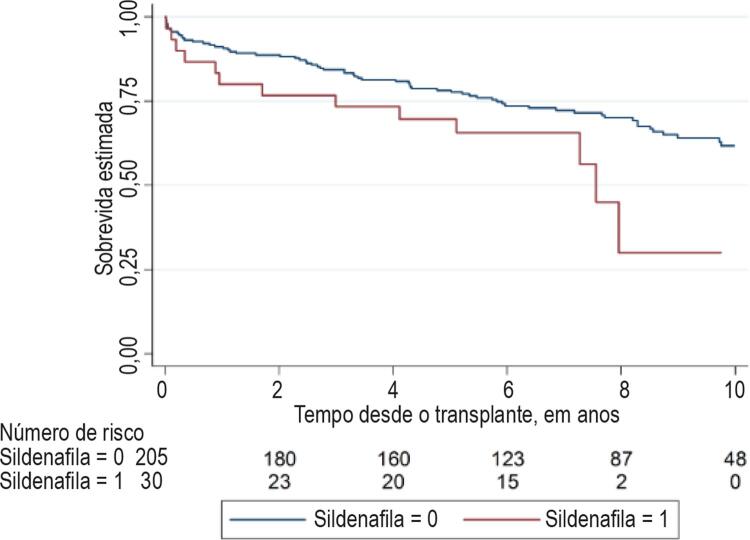
– Análise de Kaplan-Meier da mortalidade global após o transplante de acordo com o grupo de tratamento com sildenafila. Log-rank p = 0,063.

**Figura 3:**
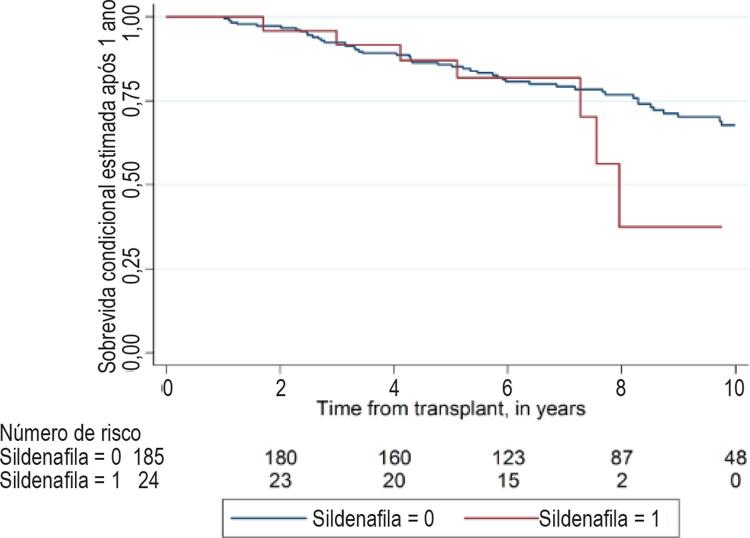
– Análise de Kaplan-Meier, análise de sobrevivência condicional após 1 ano. Log-rank p = 0,321.

A mortalidade global pós TxC é apresentada na Figura 2 (Log-rank p = 0,055). O índice de sobrevivência após o TxC do grupo A foi 97% após 30 dias, 87% após 6 meses, e 80% após um ano. No grupo B, a sobrevivência após os mesmos períodos foi de 96%, 93% e 91%, respectivamente. A diferença no ponto temporal de um ano não foi estatisticamente significativa (Log-rank p = 0,063). Depois do primeiro ano, o índice de mortalidade era similar entre os dois grupos, conforme mostrado na Figura 3 (sobrevivência condicional após 1 ano, Log-rank p = 0,321).

## Discussão

O tratamento de candidatos a TxC com HP fixa com sildenafila garantiu um período pós-operatório bem-sucedido para a maioria dos pacientes para os quais o TxC havia sido contraindicado inicialmente. Embora apresentassem hemodinâmica pior logo após o TxC, e mortalidade numericamente mais alta durante o primeiro ano, o prognóstico durante o acompanhamento de médio a longo prazo foi semelhante ao dos pacientes de TxC sem HP.

O limite entre HP fixa e reversível não é claro e não há concordância sobre o tempo necessário para se atingir o nível de irreversibilidade teórica ou sobre os melhores parâmetros para definir esse status.^12^ Em nosso centro, o CCD é utilizado rotineiramente com um teste vasodilatador, pois pose ser útil para estabelecer o risco de morte após o TxC.^5^ Uma das variáveis mais úteis para avaliar o risco é a RVP.^13^ Conforme demonstrado por Taylor et al.^19^ a RVP é um indicador independente de morte precoce após o TxC. Esse grupo relatou que o índice de sobrevivência em pacientes de TxC foi significativamente melhor quando o RVP ficava entre 1 e 3 UW, em comparação com pacientes com RVP entre 3 a 5 UW. Pacientes com RVP > 5 UW apresentaram os piores resultados. Em nosso estudo, utilizamos a sildenafila para diminuir a RVP (3,3 ± 2,3 UW), qualificando, dessa forma, os pacientes para o TxC. Na verdade, entre nossos pacientes que foram tratados com sildenafila, a RVP média era significativamente elevada e excluiria a possibilidade de TxC (5,4 ± 2,3 UW), se não fosse feita nenhuma intervenção. Se esses pacientes não fossem transplantados, seu prognóstico com tratamento médico teria sido ruim, a menos que um dispositivo de assistência ventricular (LVAD, do inglês *left ventricular assist device*) fosse implantado.

Curiosamente, dois estudos recentes sugeriram que o suporte do LVAD e a descarga mecânica não pulsante contínua do VE pode reverter uma hipertensão pulmonar que anteriormente não respondia a medicação, e tornar os pacientes aptos ao TxC.^20,21^ É interessante notar que a RVP pré-LVAD nesses estudos (4,3 ± 1,7 UW e 4,8 ± 1,8 UW) foi semelhante à do nosso estudo coorte (5,4 ± 2,3 UW). De acordo com Perez-Villa et al.^22^ uma estratégia de redução da RVP elevada usando terapia via oral (sildenafila ou bosentana) em pacientes considerados não aptos ao TxC devido à RVP elevada é viável e pode reduzir o risco de disfunção de VD pós-operatória, como também demonstramos em nosso estudo.

Os inibidores de PDE5 têm despertado o interesse no campo da doença do coração esquerdo.^6,12^Além da terapia padrão de IC, a intervenção com sildenafila pode melhorar os parâmetros hemodinâmicos pulmonares.^6,12^ Esses efeitos favoráveis surgem de sua inibição seletiva de guanosina monofosfato cíclico (GMPc) nos vasos pulmonares, que promove a vasodilatação e menos remodelagem, além de um efeito semelhante ao da milrinona no VD, devido a um processo de diafonia molecular que pode inibir o PDE3 e aumentar a contratilidade do VD.^15,18^Em uma meta-análise recente,^2^ identificou-se que o tratamento com sildenafila reduz a RVP em comparação com o uso de placebo (diferença de média ponderada -1,0 UW, p < 0,01).^2^ Nosso estudo também demonstrou que a administração de sildenafila pré-TxC em candidatos ao TxC com HP teve um efeito hemodinâmico positivo ao reduzir a RVP em aproximadamente 2 UW.

A insuficiência circulatória do lado direito é a morbidade a ela associada ainda são uma fonte importante de morte no perioperatório para pacientes de TxC. Pons et al.^12^ também avaliaram os efeito do uso continuado de sildenafila nos resultados clínicos de TxC (acompanhamento médio, 3,4 ± 2,1 anos). Neste estudo, o índice de sobrevivência após o TxC no grupo de pacientes pré-tratados com sildenafila (incluindo apenas 15 pacientes) foi de 87% após 30 dias. É importante observar que nenhum outro paciente morreu durante o período de acompanhamento de 5 anos após o TxC. Comparativamente, o índice de sobrevivência do grupo A foi 97% após 30 dias e 70% após cinco anos. Em conformidade com isso, no ISHLT* - International Registry for Heart Transplantation*, o índice de sobrevivência após 5 anos era de 72%, semelhante a nosso grupo de pacientes com HP fixa pré-tratada com sildenafila.^23^

Por todos esses motivos, uma estratégia usando a sildenafila para reduzir a RVP pode ser considerada uma “terapia de resgate” valiosa em um grupo de pacientes com IC terminal, que não estariam aptos ao TxC de outra forma. Nossos dados mostram que ela está associada a índices de mortalidade no perioperatório e em longo prazo semelhantes aos observados em pacientes sem HP.

### Limitações

As limitações deste estudo incluem sua natureza retrospectiva e não controlada, o que pode condicionar uma seleção tendenciosa. Entretanto, incluímos todos os pacientes que foram transplantados consecutivamente em nosso centro, e nenhum paciente foi perdido durante o período de acompanhamento. Além disso, o tamanho de nossa amostra é relativamente pequeno, o que limita o poder estatístico. No entanto, relatamos o que acreditamos ser a maior série existentes de pacientes de TxC pré-tratados com sildenafila. Outra limitação é a ausência de medições diretas da função do VD imediatamente após o TxC. Tentamos compensar esse fato utilizando uma medição hemodinâmica da função do VD coletada 7 dias após o procedimento. Apesar de todas essas limitações, acreditamos que os resultados podem ter validade externa para outras populações com IC avançada, já que os dados demográficos, clínicos e hemodinâmicos estão alinhados com os relatados em outros estudos.

## Conclusão

O uso de sildenafila em candidatos a TxC com HP fixa melhorou a hemodinâmica pulmonar, levando-a a um limiar em que o transplante seria possível. Nesse grupo de pacientes de alto risco, a hemodinâmica pós-operatória inicial e os resultados foram ligeiramente comprometidos, em comparação com pacientes sem HP. Entretanto, após 1 ano, os resultados de médio a longo prazo foram semelhantes entre os grupos. Nossos achados corroboram o conceito de que a sildenafila pode resgatar pacientes previamente inaptos para o TxC.
