# Supplementary material for: Resultados Clínicos e Hemodinâmicos de Longo Prazo após o Transplante de Coração em Pacientes Pré-Tratados com Sildenafil
Source: Arq Bras Cardiol. 2021 Feb 19;116(2):219–26. [Article in Portuguese] doi: 10.36660/abc.20190047 (PMC7909968; doi:10.36660/abc.20190047)
Supplement: Translation

# Translation: Long-Term Clinical and Hemodynamic Outcomes after Heart Transplantation in Patients Pre-Treated with Sildenafil

**Authors:** Sofia Lázaro Mendes, Nadia Moreira, Manuel Batista, Ana Rita Ferreira, Ana Vera Marinho, David Prieto, Rui Baptista, Susana Costa, Fatima Franco, Mariano Pego, Manuel de Jesus Antunes

**Affiliations:** 1 PortugalCentro Hospitalar e Universitario de Coimbra EPE, Coimbra - Portugal

**Keywords:** Vascular Resistance, Heart Transplantation, Hypertension Pulmonary, Sildenafil Citrate, Phosphodiesterase 5 Inhibitors, Ventricular Dysfunction, Right

## Abstract

**Background:**

Elevated pulmonary vascular resistance remains a major problem for heart transplant (HT) candidate selection.

**Objective:**

This study sought at assess the effect of pre-HT sildenafil administration in patients with fixed pulmonary hypertension.

**Methods:**

This retrospective, single-center study included 300 consecutive, HT candidates treated between 2003 and 2013, in which 95 patients had fixed PH, and of these, 30 patients were treated with sildenafil and eventually received a transplant, forming Group A. Group B included 205 patients without PH who underwent HT. Pulmonary hemodynamics were evaluated before HT, as well as 1 week after and 1 year after HT. Survival was compared between the groups. In this study, a p value < 0.05 was considered statistically significant.

**Results:**

After treatment with sildenafil but before HT, PVR (-39%) and sPAP (-10%) decreased significantly. sPAP decreased after HT in both groups, but it remained significantly higher in group A vs. group B (40.3 ± 8.0 mmHg vs 36.5 ± 11.5 mmHg, p=0.022). One year after HT, sPAP was 32.4 ± 6.3 mmHg in group A vs 30.5 ± 8.2 mmHg in group B (p=0.274). The survival rate after HT at 30 days (97% in group A versus 96% in group B), at 6 months (87% versus 93%) and at one year (80% vs 91%) were not statistically significant (Log-rank p=0.063). After this first year, the attrition rate was similar among both groups (conditional survival after 1 year, Log-rank p=0.321).

**Conclusion:**

In patients with severe PH pre-treated with sildenafil, early post-operative hemodynamics and prognosis are numerically worse than in patients without PH, but after 1 year, the medium to long-term mortality proved to be similar. (Arq Bras Cardiol. 2021; 116(2):219-226)

## Introduction

Heart transplant (HT) is the gold-standard of care for end-stage heart failure.^1^ Epidemic studies have shown that 60-70% of heart failure (HF) patients develop pulmonary hypertension (PH).^2,3^ In a Mayo Clinic study,^4^ there was a strong positive graded association between systolic pulmonary artery pressure (sPAP) and mortality, and for this reason, the presence of severe PH is one of the major contraindications to HT because of post-operative right heart dysfunction.^5^

Elevated right-sided pressures in HF usually result from elevated left ventricle (LV) filling pressures. Therefore, diastolic pulmonary artery pressure (dPAP) correlates closely with pulmonary capillary wedge pressure (PCWP).^6,7^ On the other hand, the vasoreactive component of PH develops with long-standing PH. It is characterized by vasospasm, vasoconstriction, and morphologic changes of the pulmonary vasculature.^8,9^In this case, PH persists even if the PCWP is lower after HT. Reflecting the “fixed” component of PH, the pulmonary vascular resistance (PVR) and the transpulmonary gradient (TPG) are elevated.^6^

At first, PH is reversible by systemic vasodilators, but later it becomes relatively stationary or “fixed”.^6,9,10^Elevated PVR increases mortality in the early post-HT period and remains a major problem for candidate selection.^11,12^ The inability of the transplanted heart to adapt to pre-existing significant PH usually results in right ventricle (RV) failure, which accounts for nearly 50% of all cardiac complications and up to 19% of all early postoperative deaths.^12,13^For this reason, the correct assessment that the reactivity of the pulmonary vasculature has in vasodilator therapy plays a crucial role in candidate selection. The American Heart Association guidelines define fixed PH as mean pulmonary artery pressure (mPAP) ≥ 25 mmHg and PVR ≥ 2.5 Wood units (WU) and/or TPG ≥ 12 mmHg, even after pharmacologic vasodilator testing.^14^

Sildenafil is a selective and potent inhibitor of phosphodiesterase type 5 (PDE5), which specifically degrades cyclic guanosine monophosphate, the second messenger of nitric oxide in vascular smooth muscle cells.^8,15^ Sildenafil has a favorable safety profile without oxygen desaturation or significant changes in heart rate or blood pressure.^16^ Several single-center studies have demonstrated a positive favorable hemodynamic effect of pre-HT sildenafil administration in HT candidates with PH.^12,17^ However, there is a paucity of data on the early and long-term outcomes of these high-risk patients.

The objective of this study was to compare the effect on early RV hemodynamics and mortality after HT of pre-HT sildenafil administration among patients with fixed PH who achieved HT eligibility and patients without PH. Our hypothesis is that patients with PH who received a transplant while taking sildenafil had a comparable prognosis to that of patients without PH.

## Methods

### Study Population

This retrospective, single-center, observational study included 300 consecutive patients, candidates to HT observed between November 2003 and December 2013. This population included 95 patients with fixed PH; of these, 30 patients were treated with sildenafil and eventually received transplants, forming Group A. Group B was formed by 205 patients without fixed PH who underwent HT.

In group A, sildenafil was administered orally at 20 mg tid, during a mean of 65 days (range 4 – 181 days) prior to HT. Sildenafil was well tolerated in all patients enrolled, with no serious adverse events observed.

### Data Collection

Clinical, laboratorial, and hemodynamic data were extracted using a dedicated software. All patients underwent a candidacy right heart catheterization (RHC) with a Swan-Ganz catheter via the femoral vein before sildenafil initiation; the group of patients that were exposed to sildenafil underwent a second RHC to assess the hemodynamic effect of the drug. After HT, right ventricular systolic and end-diastolic pressures were registered during the first endomyocardial biopsy, which was performed at 1 week after HT. A late hemodynamic follow-up was collected during the predefined RHC at 1 year after HT in both groups.

Cardiac output (CO) was measured by the Fick method, and cardiac index (CI) was calculated by dividing the CO by the body surface area. PCWP, sPAP, dPAP, and mPAP were measured automatically. PVR and TGP were calculated using the following formulas: TPG (mmHg) = mPAP - PCWP; PVR (WU) = TGP/CO.^18^ A follow-up was conducted for a median of 6.9 years (range 4.2 – 6.9 years) by personal interview in the outpatient ward, through a review of hospital registries, and by telephone contact, and was obtained for every patient included in this study. Confidentiality was always respected.

### Endpoints

The co-primary outcome measures were (1) RV systolic pressure and end-diastolic pressure (the latter used as a surrogate of RV function) at 7 days after HT and (2) the sPAP and PVR 1 year after HT; the secondary outcome was the all-cause mortality after HT. The endpoints were compared between the pre-defined groups.

### Statistical Analysis

Continuous variables were normally distributed and assessed using the Shapiro-Wilk test, and expressed as means±standard deviations, while those with non-normal distribution were expressed as median (interquartile range). Dichotomous variables were expressed as frequencies (percentages). To compare data between the groups, the Student`s T-test (Unpaired T-test) for continuous variables, the Mann-Whitney test for non-continuous data, and the Chi-Square test (Fisher, as appropriate) for dichotomous data were used. The McNemar test was used for paired categorical analysis. Kaplan-Meyer survival curves were constructed and compared using the Log-rank test. Conditional survival was assessed by limiting the group of patients analyzed to those who have survived to at least 1 year. The entire analysis was performed using STATA 12.0 (College Station, Texas, USA). Graphs were constructed with GraphPad 5.0 (La Jolla, California, USA). In this study, a p value < 0.05 was considered statistically significant.

## Results

All 235 patients underwent successful HT. Baseline characteristics are presented in Table 1. Most patients were male, and the mean age of group A was 53.6 ± 10.9 years and of group B 52.9 ± 13.4 years (p = 0.545). Pre-HT hemodynamics are presented in Table 2 and were significantly different among groups. Group A patients displayed more severe pulmonary hemodynamics that Group B patients. After treatment with sildenafil but before HT, PVR (-39%) and sPAP (-10%) decreased significantly (Table 3).

**Table 1:** – Characteristics of Patients with (Group A) and without (Group B) Sildenafil Pre-Treatment Before Heart Transplant

Characteristic^**a**^	Group A (n=30)	Group B (n = 205)	p-value^**b**^
Mean Age, years	53.6 ± 10.9	52.9 ± 13.4	0.545
Gender Male, %	86.7	76.2	0.247
**Etiology**			
Ischemic, %	50.0	34.0	0.346
Idiopathic, %	36.7	56.3	
Hypertrophic, %	3.3	4.4	
Restrictive, %	10.0	2.9	
Congenital, %	0.0	2.4	
**NYHA Class**			
III, %	33.3	36.4	0.968
IV, %	66.7	63.6	
**Laboratorial Parameters**			
Hemoglobin, g/dl	12.3 ± 1.8	12.7 ± 1.7	0.815
Creatinine, mg/dl	1.4 ± 1.0	1.3 ± 0.5	0.060
BNP, pg/ml	524 [396 - 912]	625 [306 - 1039]	0.906
**Cardiac Parameters**			
LVEF, %	19.6 ± 4.5	21.2 ± 8.4	0.021
**Mitral Regurgitation**			
Mild, %	16.0	12.5	0.703
Moderate, %	32.0	34.0	
Moderate-Severe, %	24.0	14.6	
Severe, %	24.0	31.2	
**Cardiac Devices**			
ICD, %	40.0	21.8	0.128
CRT, %	10.0	22.4	
**Sildenafil, pre-HTx**			
Duration, days	65 [4 – 181]		

BNP: blood natriuretic peptide; CRT: cardiac resynchronization therapy; HT: heart transplant; ICD: implantable cardioverter defibrillator; LVEF: left ventricular ejection fraction; NYHA: New York Heart Association. ^*a*^Data are expressed as percentages, mean ± standard deviation or median (interquartile range). ^*b*^Student`s T-test for continuous variables with normal distribution, Mann-Whitney test for continuous variables without normal distribution and Chi-Square test for categorical variables.

**Table 2:** – Hemodynamic Variables before Heart Transplant in Patients with (Group A) and without (Group B) Severe Hypertension

Variable	Group A Means ± SD	Group B Means ± SD	p-value^** a**^
PVR, WU	5.4 ± 2.3	2.7 ± 1.8	< 0.001
**PAP, mmHg**			
Systolic	58.9 ± 16.4	44.5 ± 15.2	< 0.001
Diastolic	23.1 ± 8.2	19.4 ± 8.0	0.025
Mean	36.4 ± 10.7	29.0 ± 10.3	0.001
CO, liters/min	3.7 ± 1.2	3.6 ± 1.0	0.645
**BP, mmHg**			
Systolic	75.0 ± 12.2	74.9 ± 10.8	0.980
HR, ppm	76 ± 18	76 ±16	0.873

BP: blood pressure; HR: heart rate; CO: cardiac output; PAP: pulmonary artery pressure; PVR: pulmonary vascular resistance; SD: standard deviation. ^*a*^Student`s T test was used.

**Table 3:** – Hemodynamic Variables before and after Heart Transplant in Patients with (Group A) and without (Group B) Sildenafil Pre-Treatment

	Eligibility RHC		3-month post-sildenafil RHC		7^**th**^-day post-HTx EMB		1-year RHC	
	sPAP (mmHg)	PVR (WU)	sPAP (mmHg)	PVR (WU)	RV systolic pressure (mmHg)	RV end-diastolic pressure (mmHg)	sPAP (mmHg)	PVR (WU)
No sildenafil	44.5 (15.2)	2.7 (1.8)	--	--	36.5 (11.5)	7.0 (7.1)	30.48 (8.23)	1.8 (1.0)
Sildenafil	58.9 (16.4)	5.4 (2.3)	52.8 (17.1)^c^	3.3 (2.3)^d^	40.3 (8.0)	7.9 (5.8)	32.43 (6.39)	1.8 (0.8)
p - value^a^	< 0.001	< 0.001	--	--	0.022^b^	0.374^b^	0.274	0.789

EMB: endomyocardial biopsy; PVR: pulmonary vascular resistance; RHC: right heart catheterization; WU: wood units. ^*a *^Student’s t-test comparing no sildenafil patients vs. sildenafil-treated patients. ^*b*^McNemmar test. ^*c*^P = 0.845 vs. no sildenafil patients. ^*d*^P = 0.806 vs. no sildenafil patients.

### Peri-HT Data and Post-HT Outcomes

The co-primary endpoint measures, assessed 1 week after HT, are presented in Table 3. The evolution of sPAP during the follow-up time in both groups is shown in Figure 1. sPAP decreased after HT in both groups, but remained significantly higher in patients pre-treated with sildenafil vs. patients that were not pretreated (40.3 ± 8.0 mmHg vs 36.5 ± 11.5 mmHg, p = 0.022). No differences were found regarding RVEDP at one week after HT, used as a surrogate of early RV dysfunction (Table 3). One year after HT, sPAP was 32.4 ± 6.3 mmHg in group A vs 30.5 ± 8.2 mmHg in group B (p = 0.274) (Table 3). PVR was also similar in the two groups (1.8 ± 0.8 mmHg versus 1.8 ± 1.0 WU, p = 0.789).

**Figure 1:** – (Left panel) Systolic pulmonary artery pressure (sPAP, in mmHg) at four different time-points: baseline before HT without sildenafil treatment, before HT with sildenafil treatment, early after HT (7 days) and late after HT (one year). ***p < 0.001, * p = 0.022. (Right panel) Pulmonary vascular resistance (PVR, in Wood units) at three different time-points: baseline before HT without sildenafil treatment, before HT with sildenafil treatment and late after HT (one year). *** p < 0.001, * p = 0.789. EMB: endomyocardial biopsy; HT: heart transplant; RHC: right heart catheterization.

### Survival Analysis

Post-HT all-cause mortality is shown in Figure 2 (Log-rank *P* = 0.055). The survival rate after HT in group A was 97% at 30 days, 87% at 6 months, and 80% at one year. In group B, survival in the same time frames was 96%, 93%, and 91%, respectively. The difference at the one-year time-point was not statistically significant (Log-rank p = 0.063). After this first year, the attrition rate was similar between both groups, as shown in Figure 3 (conditional survival after 1 year, Log-rank p = 0.321).

**Figure 2:** – Kaplan-Meier analysis of all-cause mortality after transplant according to sildenafil treatment group. Log-rank p = 0.063.

**Figure 3:** – Kaplan-Meier analysis one-year conditional survival analysis. Log-rank p = 0.321.

## Discussion

Treatment of HT candidates with fixed PH with sildenafil enabled a successful post-operative period for most of the patients that were initially contraindicated for HT. Although displaying poorer hemodynamics shortly after the HT, and a numerically higher mortality during the first year, the prognosis during medium to long term follow-up was similar to that of HT patients without PH.

The limit between fixed and reversible PH is unclear, and there is no agreement on the time needed to reach the level of theoretical irreversibility and the best parameters to define this status.^12^ At our center, RHC is routinely used with a vasodilator test, as this may be useful to establish the risk of death after HT.^5^ One of the most useful variables to asses this risk is the PVR.^13^ As shown by Taylor et al.^19^ PVR is an independent predictor of early death after HT. This group reported that the survival in HT patients was significantly better if PVR was between 1 to 3 WU, compared with recipients with a PVR 3 to 5 WU, while patients with PVR > 5 WU had the worst outcomes. The present study used sildenafil to decrease PVR (3.3 ± 2.3 WU), thus making the patients eligible for HT. In fact, among the patients that were treated with sildenafil, the average PVR was significantly elevated and would preclude HT (5.4 ± 2.3 WU) if no intervention had been done. Moreover, if these patients were not transplanted, their prognosis under medical therapy would have been poor unless a left ventricular assist device (LVAD) were implanted.

Interestingly, two recent studies suggest that LVAD support and continuous nonpulsatile mechanical unloading of the LV can reverse a previously medically unresponsive pulmonary hypertension and render patients eligible for HT.^20,21^ Of interest, pre-LVAD PVR in these studies (4.3 ± 1.7 WU and 4.8 ± 1.8 WU) was similar to that of our cohort (5.4 ± 2.3 WU). According to Perez-Villa et al.^22^ a strategy of reducing elevated PVR using oral therapy (sildenafil or bosentan) in patients considered ineligible for HT because of elevated PVR is feasible and may reduce the risk of post-operative RV dysfunction, as we have also shown in our study.

PDE5 inhibitors are receiving increasing interest in the field of left heart disase.^6,12^ In addition to standard HF therapy, sildenafil intervention might improve the pulmonary hemodynamic parameters.^6,12^ These favorable effects arise from its selective inhibition of the hydrolysis of cyclic guanosine monophosphate (cGMP) in the pulmonary vasculature, which promotes vasodilation and less remodeling, as well as a milrinone-like effect in the RV due to a process of molecular crosstalk that can inhibit PDE3 and increase RV contractility.^15,18^In a recent meta-analysis,^2^ sildenafil treatment was found to reduce PVR compared with placebo (weighted mean difference -1.0 WU, p < 0.01).^2^ Our study also demonstrated that pre-HT sildenafil administration in HT candidates with PH had a positive hemodynamic effect by reducing PVR by about 2 WU.

Right-sided circulatory failure and its associated morbidity remains an important source of peri-operative death for HT patients. Pons et al.^12^ also evaluated the effects of chronic sildenafil use on clinical outcomes in HT (mean follow-up, 3.4 ± 2.1 years). In this study, the survival rate after HT in the group of patients pre-treated with sildenafil (including only 15 patients) was 87% at 30 days. Importantly, no other patient died during the 5-year follow-up period after HT. By comparison, the survival rate after HT in group A was 97% at 30 days and 70% at five years. Accordingly, in the ISHLT International Registry for Heart Transplantation, the survival rate at five years was 72%, similar to our group of patients with fixed PH pre-treated with sildenafil.^23^

For all these reasons, a strategy of using sildenafil to reduce PVR can be considered to be a valuable “rescue therapy” in a group of patients with end-stage HF, who would otherwise not be eligible for HT. Our data show that it is associated with similar perioperative and long-term mortality similar to that observed in patients without fixed PH.

### Limitations

The limitations of this study include its retrospective and uncontrolled nature, potentially conditioning selection bias. However, we included all patients that were consecutively transplanted in our center and no patient was lost to follow-up. In addition, the size of our sample is relatively small, limiting the statistical power. However, to the best of our knowledge, to date, this is the largest case series on HT patients pre-treated with sildenafil. Another limitation is the absence of direct RV function measurements immediately after HT; we tried to compensate for this fact using a hemodynamic measurement of RV function collected 7 days after the procedure. Despite all these shortcomings, we believe that the results can have external validity for other advanced HF populations, as the demographic, clinical, hemodynamic, and prognostic data are in line with those reported in other trials.

## Conclusion

The use of sildenafil in HT candidates with fixed PH improved pulmonary hemodynamics to a threshold where transplant was possible. In this high-risk group of patients, early post-operative hemodynamics and results were slightly compromised when compared with patients without PH. However, after 1 year, the medium to long-term outcomes were similar between the groups. Our findings support the concept that sildenafil can rescue previously ineligible patients for HT.
